# Parents' Perspectives Toward School Reopening During COVID-19 Pandemic in Indonesia—A National Survey

**DOI:** 10.3389/fpubh.2022.757328

**Published:** 2022-04-04

**Authors:** Antonius Hocky Pudjiadi, Nina Dwi Putri, Hikari Ambara Sjakti, Piprim Basarah Yanuarso, Hartono Gunardi, Rosalina Dewi Roeslani, Ade Djanwardi Pasaribu, Lies Dewi Nurmalia, Catharine Mayung Sambo, Lathiefatul Habibah, Indriyanti Natasya Ayu Utami, Yogi Prawira, Nastiti Kaswandani, Anggraini Alam, Kurniawan Taufiq Kadafi, Gryselda Hanafi, Angela Kimberly Tjahjadi, Shindy Claudya Aprianti, Nabila Maudy Salma, Stephanie Wijaya, Fatima Safira Alatas, Aman Bhakti Pulungan

**Affiliations:** ^1^Indonesian Pediatric Society, Jakarta, Indonesia; ^2^Department of Child Health, Cipto Mangunkusumo National Central Hospital, Faculty of Medicine Universitas Indonesia, Jakarta, Indonesia; ^3^Department of Pediatrics, Hasan Sadikin Hospital, Faculty of Medicine Universitas Padjajaran, Bandung, Indonesia; ^4^Department of Pediatrics, Dr. Saiful Anwar Hospital, Faculty of Medicine Universitas Brawijaya, Malang, Indonesia

**Keywords:** school reopening, parents, survey, COVID-19, pandemic

## Abstract

**Background:**

All sectors are affected due to COVID-19 pandemic occurring worldwide, including the education industry. School closure had been taking place for more than a year in Indonesia. Despite the controversies, Indonesian government had decided to begin school reopening.

**Objectives:**

This study aims to assess parental readiness for school reopening, and factors affecting parental attitude toward school reopening.

**Methods:**

A cross-sectional study using online questionnaire distributed *via* official Indonesian Pediatric Society (IPS) official social media account collected between March and April 2021. The questionnaire contained the general characteristics of study participants, parents' knowledge, and perspectives on COVID-19, and health protocols for school reopening.

**Results:**

A total of 17,562 responses were collected, of which 55.7% parents were ready to send their children to school should school reopens. Factors significantly contribute to parental decision to keep their child at home were: presence of vulnerable population at home [OR = 1.18 (1.10–1.27), *p* < 0.001], children with comorbidities [OR = 2.56 (2.29–2.87), *p* < 0.001], perception of COVID-19 as a dangerous disease [OR = 28.87 (14.29–58.33), *p* < 0.001], experience with COVID-19 positive cases in the community [OR = 1.75 (1.61–1.90), *p* < 0.001], COVID-19 related death in the community [OR = 2.05 (1.90–2.21), *P* < 0.001], approval for adult COVID-19 vaccination [OR = 1.69 (1.53–1.87), *p* < 0.001], and ownership of private transportation [OR = 1.46 (1.30–1.66), *p* <0.001].

**Conclusion:**

We identified several factors affecting parental perception on school reopening during COVID-19 pandemic that should be addressed. This study can be used for policy-maker to make further recommendations and health educations prior to school reopening in Indonesia.

## Introduction

The outbreak of coronavirus disease 2019 (COVID-19) was declared a pandemic more than 1 year ago (1). It is a systemic disease that mainly affects the respiratory system and is caused by the novel virus severe acute respiratory syndrome coronavirus-2 (SARS-CoV-2) (2). The outbreak was first reported in Wuhan, Hubei province, China, at the end of 2019. The route of transmission is mainly through close contact and respiratory droplets (2). As of June 6, 2021, the number of new active confirmed cases of COVID-19 disease reported was 3,016,005 (cumulative cases: 172,637,970). The five countries that had the most cases were India, Brazil, Argentina, Colombia, and the United States of America. In the Asiatic region, Indonesia had the second-highest number of cases after India (3).

In March 2020, the Indonesian government announced its first two cases of COVID-19 (4). Since then, 1,856,038 confirmed cases, 5,832 daily active cases, and 51,612 deaths (case fatality rate of 2.78%) were reported across the country as of June 6, 2021. Notably, 12.6% of all positive cases and 1.2% of deaths were in the pediatric population (4). Cumulatively, the highest proportions of COVID-19 cases among school-aged children occurred in those of elementary school age (7–12 years old; 28%), senior high school age (16–18 years old; 25.2%), and junior high school age (13–15 years old; 19.9%). The case fatality rate among children with COVID-19 was 0.27% (4).

In response to the health emergency caused by the pandemic, adjustments were made to all sectors, including education (5). In March 2020, the Ministry of Education (MOE) distributed guidelines about implementation of education policies during the COVID-19 pandemic (6). All schools throughout Indonesia were closed, and students were urged to carry out virtual learning at their respective homes. Conventional learning was only allowed to be conducted in green and yellow zones (see Indonesia's zoning guidelines in Supplementary Material 1, Supplementary Table 1), which were deemed to have a considerably lower risk of COVID-19 transmission than other areas (6–8). However, prolonged virtual learning is associated with an increase in the risk of school dropout, a decrease in overall academic ability, and an increase in domestic violence, especially in lower-income households. Many children were forced to work due to economic difficulties during the COVID-19 pandemic, and some households even chose arranged marriage as a way to escape poverty (9, 10).

After ~1 year of virtual learning, the MOE, together with the Ministry of Religion, the Ministry of Health (MOH), and the Ministry of Home Affairs, finally issued a Joint Ministerial Decree on school reopening in all zones (11). In addition, the Indonesian Pediatric Society (IPS) released recommendations that considered the children's rights, the current development of the pandemic, and the COVID-19 vaccination coverage in Indonesia (12). According to these guidelines (joint decree on school reopening), school reopening is allowed only if the positivity rate in the area is below 5% and the mortality rate is declining. In addition, schools must complete the school readiness checklists issued by the IPS before the initiation of conventional learning (12). If any positive cases are identified, the school must be closed for a minimum of 3 days. Moreover, parents must be given the choice of whether to send their children back to school or continue virtual learning, and schools must guarantee equal rights and obligations for virtual and conventional learning (6). Both the MOH and the IPS stressed the importance of COVID-19 vaccinations among school employees, adequate ventilation of classrooms, and physical distancing when schools reopen (6, 12).

Currently, Indonesia is preparing for school reopening due to declining cases of COVID-19 (13). The government is actively promoting vaccinations, especially for particularly susceptible populations and those who are essential workers. This is the first time since the pandemic started that all schools will resume conventional learning. School reopening requires careful risk assessment, including analysis of the preparedness of the local health protocols and facilities as well as the preparedness of the people involved (students, school officers, and parents). This study was conducted to better understand parents' readiness for school reopening. It is important to know parents' concerns, as they are the primary caretakers of the children, and by understanding their concerns and the problems that may arise, stakeholders can make adjustments and provide the information needed to better prepare for school reopening.

## Methods

This was a cross-sectional conducted using a digital questionnaire about COVID-19 school reopening. The questionnaire was adopted and modified from several resource and adjusted to local context. All members of The Indonesian COVID-19 Task Force Expert Panel of the IPS approved the content of the questionnaire. An invitation post for a digital survey (Google Form) was advertised through the IPS' social media accounts. Participants could access the digital survey remotely with their electronic devices. Parents who had children attending preschool, kindergarten, primary school, secondary school, and/or high school were included in the study. The questionnaire contained four sections: (a) general characteristics of study participants (children's age, children's education level, and presence of individuals with comorbidities or in a vulnerable population at home); (b) parents' knowledge about COVID-19 (transmission, risk factors, willingness to receive COVID-19 vaccinations, source of information, and reliability of information); (c) perspectives about school reopening in Indonesia (regulation, concerns, and adjustments in the learning process); and (d) health protocols for school reopening. This questionnaire was developed in the local language (Bahasa Indonesia) based on the IPS' recommendations. The first page of the survey contained an informed consent form, the objectives of the study, and assurance of data confidentiality. If the participant agreed to participate, then they could continue to fill in the questionnaire; otherwise, they were redirected to the final page of the survey. Multiple choice questions were presented, and all questions had to be answered to finish the questionnaire. Data collection was conducted between March 9 and April 30, 2021, and the total population sampling method was used (all respondents were included in the analysis). The survey responses were stored in a private database that could only be accessed by the authors. The study was reviewed and approved by the Ethics Committee of the Faculty of Medicine, Universitas Indonesia (Letter No: KET-634/UN2.F1/ETIK/PPM.00.02/2021).

### Statistical Analysis

SPSS version 25.0 was used to perform statistical analysis in this study. Categorical data are presented as frequencies in tables and figures, analyzed using Chi-square test or Fisher's exact test as appropriate. A *p*-value of <0.05 for two-sided hypothesis was considered statistically significant. Variables with a *p*-value of <0.25 were included in the logistic regression multivariate analysis. Participants who chose “equally likely” in response to the question about whether they would send their child to conventional school or continue virtual learning were excluded in the multivariate analysis. Stepwise backward elimination was performed for all variables and the data was presented in the form of adjusted odd's ratio (OR) and 95% confidence interval (CI).

## Results

A total of 17,562 participants were included in the analysis. The median age of the children of participants was 9 years (IQR 7–12). General characteristics of the study participants are summarized in Table 1. Respondents hailed from all 34 provinces of Indonesia, comprising individuals from Java (52.4%), Kalimantan (4.8%), Sulawesi (3.3%), Sumatra (3.0%), Lesser Sunda Island (0.5%), Maluku Island (0.2%), and Western New Guinea (0.2%). The remaining respondents (35.7%) did not fill in the province of origin.

**Table 1:** General characteristics of respondents' children (*n* = 17,562).

**Parameters**	**Frequency (%)**
Child's age in years, median (interquartile range)	9 (7–12)
Education level of the child, *n* (%)	
Preschool (3–4 years)	518 (2.9)
Kindergarten (4–6 years)	2,606 (14.8)
Primary school (7–12 years)	9,786 (55.7)
Secondary school (13–15 years)	2,916 (16.6)
High school (16–18 years)	1,736 (9.9)
Vulnerable individual(s) in the household, *n* (%)	
Yes	11,540 (65.7)
No	6,022 (34.3)
Children with chronic illness or comorbidities, *n* (%)	
Yes	1,651 (9.4)
No	15,911 (90.6)
Parents' choice about whether to send their child to school, *n* (%)	
Send to school	9,774 (55.7)
Keep at home	6,368 (36.3)
Equally likely	1,420 (8.1)

**Figure 1:**
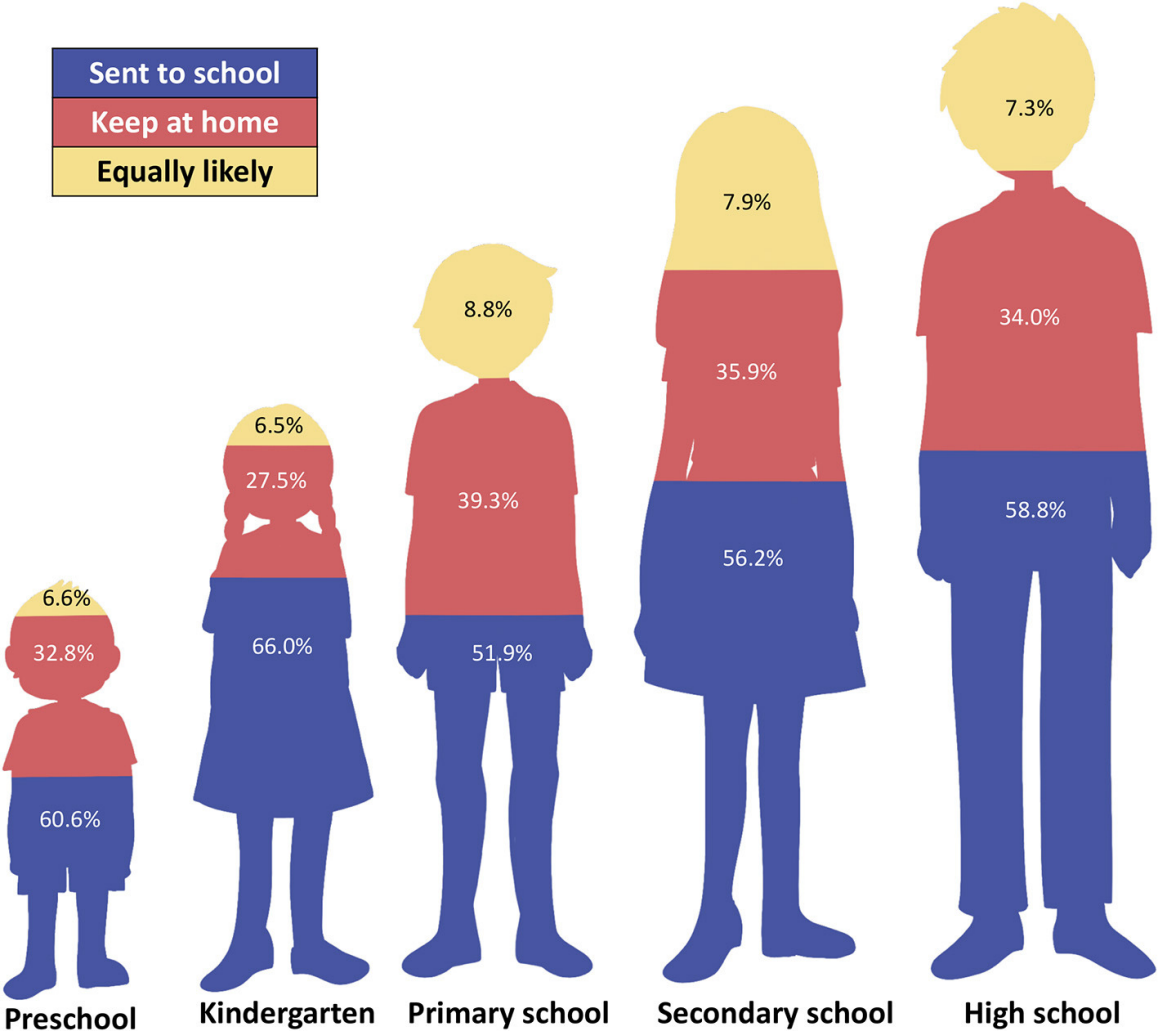
Parents' attitude toward school reopening according to children's education level.

The parents' preferred type of learning during the pandemic is shown in Figure 1. Regardless of their children's age, more than 50% of respondents answered that they would send their children to school after school reopening, with 66.0% of parents of kindergarten students, 60.6% of parents of preschool students, and 58.8% of parents of high school students stating that they would send their children to school. Parents were concerned that the amount of time in school should be reduced or preferably to be split in order to prevent overcrowding (87%). Fifty-one percent of participants supported alternating school days between conventional and virtual classes.

Most parents accessed information from digital media such as TV or radio (78.6%) and social media such as Instagram, Facebook, or WhatsApp groups (70.1%). The COVID-19 information they received was considered clear and comprehendible by 89.2% of the subjects. Overall, the respondents had a median score of four out of five on confidence toward source of COVID-19 information. Knowledge of COVID-19 was assessed to gauge the accuracy of information obtained by parents (Table 2). Almost 95% of subjects knew that asymptomatic COVID-19 patients could be infectious. Incorrect statements (e.g., COVID-19 is a mild bacterial infection) were chosen by only 4% of 17,562 respondents. Most respondents thought that SARS-CoV-2 among children can be transmitted through droplets generated during coughing or sneezing (85.3%) and touching the face after direct contact with contaminated items (83.1%). A total of 91% of participants agreed that everyone, including infants, children, adults, and geriatrics, could transmit and contract COVID-19. Fifty-nine percent of subjects considered COVID-19 to be an extremely dangerous ailment (score 5 out of 5). The number of subjects who were willing to have themselves and their children vaccinated was 84.4 and 78.5%, respectively.

**Table 2:** Parents' knowledge about COVID-19.

**Questions (*n* = 17,562)**	**Response, *n* (%)**
What do you think COVID-19 is? (multiple answers will be accepted)	
a.It is a disease caused by the novel coronavirus	15,116 (86.1)
b.It is a mild bacterial infection	795 (4.5)
c.It is a pandemic affecting the entire world	9,835 (56.0)
d.It is a minor disease that cannot cause death	362 (2.1)
What is the method of transmission for COVID-19 in children? (multiple answers will be accepted)	
a.Through respiratory droplets when people cough or sneeze	14,983 (85.3)
b.Direct contact, such as touching objects or shaking hands that are contaminated with the virus and then touching the face area before washing hands	14,599 (83.1)
c.Through air contaminated with coronavirus in an enclosed place	691 (3.9)
d.Through sweat	1,407 (8.0)
e.Through sexual intercourse	1,048 (6.0)
Who can transmit and contract COVID-19? (multiple answers will be accepted)	
a.Everyone from babies to older people	16,060 (91.4)
b.Vulnerable people only (example: those with comorbidities)	941 (5.4)
c.Everyone except babies and children	561 (3.2)
Can people who have COVID-19 and are asymptomatic transmit the disease?	
a.Yes, they can transmit	16,619 (94.6)
b.No, they cannot transmit	569 (3.2)
c.Not sure	374 (2.1)

This study found several factors that influence parents' perspectives toward school reopening during the COVID-19 pandemic, including the presence of a vulnerable individual(s) in their home, children with comorbidities, confirmed COVID-19 cases, death caused by COVID-19 in the community, opinion on vaccination of parents and children and preferred mode of transportation, as shown in Table 3. In summary, 9,774 (55.7%) respondents agreed to send their children to school when schools reopen, while 6,369 (36.3%) disagreed. The remaining respondents did not give a decisive answer (7.7%). Multivariate analysis of factors associated with parents' attitudes toward in-school learning was conducted to understand which factors contributed the most in the decision-making process. The most influential factor associated with keeping children in virtual learning was the opinion that COVID-19 is a dangerous disease (adjusted OR = 23.57; 95% CI: 11.56–48.04). Multinomial analysis including respondents who choose equally likely to send their children to school is attached in Supplementary Material 2.

**Table 3:** Final multivariate analysis of factors associated with parents' attitudes toward in-school or virtual learning (*n* = 16,142).

**Variables**	**Send to school** **(ref); *n* (%)**	**Keep at home;** ***n* (%)**	***p*** **-value**	**Adjusted OR (95% CI)**
Vulnerable population in the household			<0.001*	1.18 (1.10–1.27)
None (ref)	3,500 (21.7)	2,033 (12.6)		
Yes	6,274 (38.9)	4,335 (26.9)		
Children with comorbidities			<0.001*	2.56 (2.29–2.87)
None (ref)	9,186 (56.9)	5,440 (33.7)		
Yes	588 (3.6)	928 (5.7)		
How dangerous is COVID-19?^a^			<0.001*	28.87 (14.29–58.33)
Mild (ref)	510 (3.2)	8 (0.0)		
Dangerous	9,264 (57.4)	6,360 (39.4)		
Any positive COVID-19 cases in community			<0.001*	1.75 (1.61–1.90)
None (ref)	4,253 (26.3)	1,441 (8.9)		
Yes	5,521 (34.2)	4,927 (30.5)		
Any death caused by COVID-19 in community			<0.001*	2.05 (1.90–2.21)
None (ref)	6,981 (43.2)	3,004 (18.6)		
Yes	2,793 (17.3)	3,364 (20.8)		
Agree COVID-19 vaccination for parents			<0.001*	1.69 (1.53–1.87)
No (ref)	1,864 (11.5)	635 (3.9)		
Yes	7,910 (49.0)	5,733 (35.5)		
Mode of transportation^b^			<0.001*	1.46 (1.30–1.66)
Public transport (ref)	648 (4.0)	606 (3.8)		
Private transport	9,126 (56.5)	5,762 (35.7)		

*ref, reference group*.

* *Significant p-value*,

a *“Mild” is score 1–3, while “dangerous” is 4–5 with 5 as the most dangerous score*.

a *Public transport include online transport and mass public transport, while private transport nclude private car, private motorbike, walking, and cycling*.

Half of all subjects (51.5%) stated that providing materials required for virtual learning, such as computers and internet connection, as well as the significantly decreased duration of learning activities, was burdensome. The main challenges and recommendations regarding virtual learning are shown in Table 4.

**Table 4:** Respondents' reported challenges and recommendations in implementing virtual learning (*n* = 17,562).

**Challenges**	**Recommendations**
° Virtual learning negatively impact my child's academic performance (47.2%)	° Interactive live discussions between teachers and students (79.4%)
° Concerned about my child's mental health (41.9%)	° Good internet connection and computers (52.9%)
° Absence of caregiver (34.2%)	° Instructional video materials (50.8%)
° Limited supporting materials such as computer or internet access (31.8%)	° Printed guidebook (36.8%)

The interactions between respondents and schools are shown in Table 5. Psychological support was regarded as crucial by 68% of participants, particularly for children who feared social interactions during this pandemic. A total of 96% of subjects demanded that the school minimize the risk of SARS-CoV-2 transmission during regular offline classes and issue clear regulations for student drop-off and pick-up.

**Table 5:** The interactions between parents and schools during online school.

**Parameters (*n* = 17,561)**	**Response; *n* (%)**
**Frequency of communication between schools and parents**	
Very often	4,138 (23.6%)
Often	9,482 (54.0%)
Seldom	3,624 (20.6%)
Never	317 (1.8%)
**Media for communication between schools and parents**	
Social media groups	16,883 (96.1%)
Phone	2,886 (16.4%)
Email	2,721 (15.5%)
Short message services	605 (3.4%)
Others	1,211 (6.9%)
**Parents' response on school preparation before reopening**	
Routine body temperature measurement of students and teachers	13,974 (79.6%)
Routine swab test for students and teachers	9,747 (55.5%)
Mandatory mask use in the school area	16,110 (91.7%)
Adequate ventilation in classrooms	13,523 (77.0%)
Limiting the number of students in each classroom	14,218 (81.0%)
Social distancing during recess	10,739 (61.2%)
Bringing packed meals to school	12,942 (73.7%)

**Figure 2:**
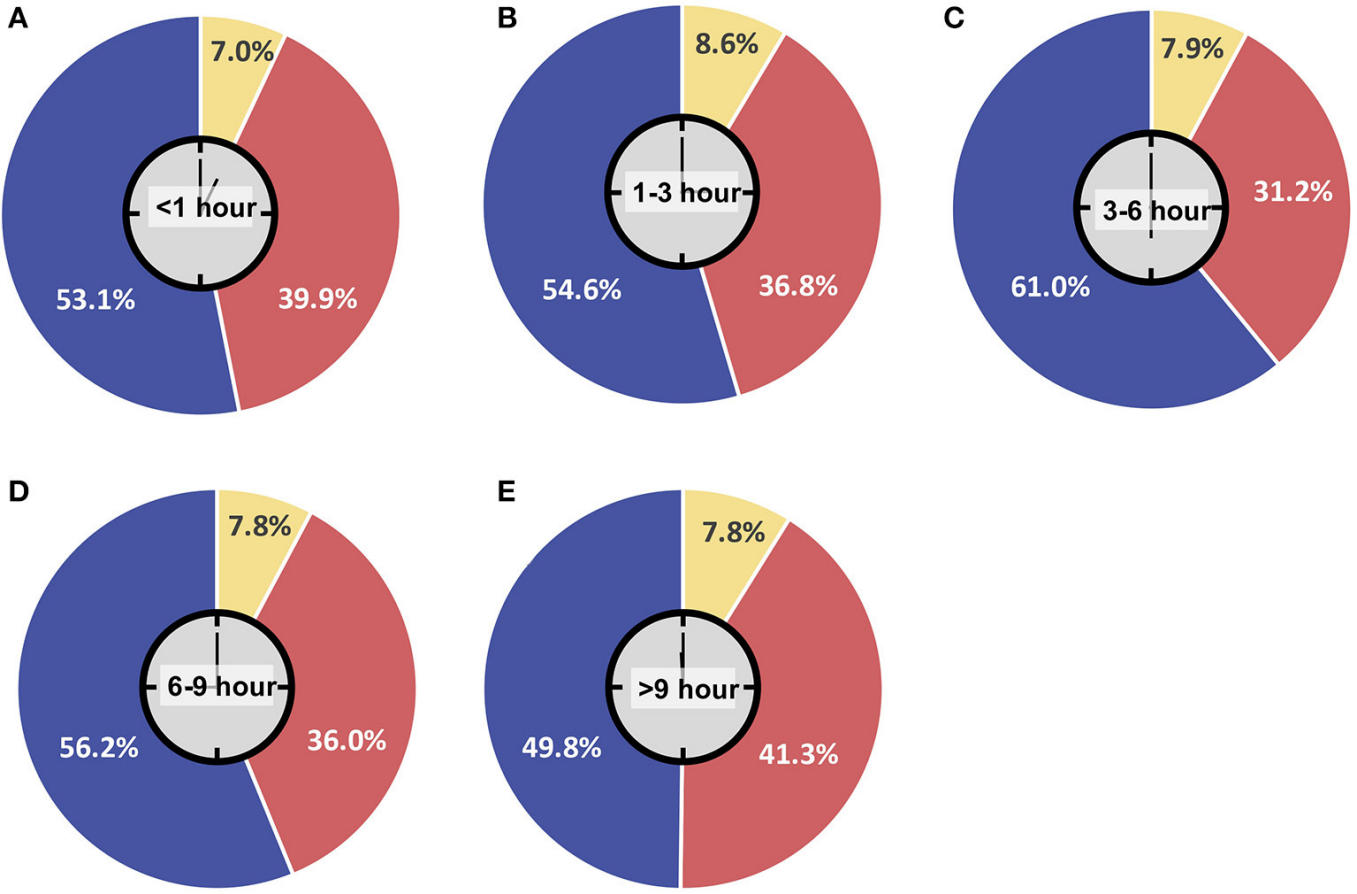
Mask-wearing tolerance among respondents' children. **(A)** Less than 1 h; **(B)** 1–3 h; **(C)** 3–6 h; **(D)** 6–9 h; **(E)** More than 9 h.

Parents' assessment of their children's ability to wear masks is shown in Figure 2. Most children were deemed able to use a mask for up to 9 h. However, similar response on decision on whether to send their children to school were found regardless of the amount of time their children can wear masks. Among parents who agreed to send their children to school, the average mask-wearing tolerance was 3–6 h. Additionally, most children were deemed able to use masks without eating or drinking for either 1–3 h (48.8%) or <1 h (36.1%). The rest (15.2%) were considered able to wear masks for more than 3 h.

## Discussions

Despite rigorous efforts in contact tracing and controlling transmission of SARS-CoV-2, the number of confirmed COVID-19 cases is still rising in Indonesia (8). Since the COVID-19 outbreak reached Indonesia in March 2020, schools, considered environments with a high risk of transmission, were closed until further notice. The government decided to resume conventional learning by July 2021 after 1 year of virtual learning (11). Most of the children of the study participants were primary school students (55.7%), with a median age of 9 years. Those who agreed to send their children to school (rather than continue virtual learning) primarily had younger children (preschool and kindergarten). This is as opposed to the stages of cognitive development in children. After the age of 11, children can exhibit abstract operational thinking, which marks the start of children's ability to make more rational decisions (14). Hence, generally, older children, especially those in secondary school, may better understand the reasons for implementation of the health protocols and exhibit higher adherence to rules and instructions.

Similar to homeschooling, virtual learning demands a commitment to self-directed studying (15). However, younger students still need of guidance to maintain the flow of their studies. A previous study among Chinese parents with young children who underwent online studying during the COVID-19 pandemic found that parents tended to experience difficulties during online learning (16). Most parents did not like online learning, as they felt that their children had more difficulty concentrating, there was a lack of self-regulation among their children, and they faced difficulties in supporting their children's online learning due to lack of time and experience. Parents with full-time jobs and those who were unable to afford daycare or caregiver support preferred conventional learning (16, 17). This might explain why some parents were inclined to send their children to school.

Nevertheless, whether to resume conventional classes was still under debate due to the high risk of viral transmission. The government's decree requires that schools fulfill a readiness checklist and parental consent is obtained before reopening conventional schools (12, 18). Adequacy, transparency, and accessibility of information certainly affect parents' confidence in and satisfaction with school reopening. Indonesians primarily relied on regular official press releases on digital media (TV or radio) and social media for information about the pandemic (19). Medical hoaxes, which may lead to confusion and excessive fear of COVID-19, had to be resolved quickly in order to gain trust from the public. Given the trust in the government and school credibility regarding school reopening practices, according to IPS and Joint Ministerial Decree recommendations, many students are expected to return to conventional schools. This expectation was supported by the results of our study, which revealed the high readiness level (median of 4 out of 5, with 5 as most ready) of parents regarding school reopening and the finding that communication between parents and schools was well maintained during the pandemic. Kroshus reported that planning to keep children home was negatively associated with confidence in schools (*B* = −0.22; *p* < 0.001) (20). Thus, comprehensibility and high trust regarding COVID-19 information were significantly associated with the decision to send children to school.

In our study, most parents who disagreed with vaccinating their children and/or themselves still agreed to school reopening. This might occur due to controversy about the efficacy of COVID-19 vaccination, fear of adverse effects of vaccination, and the existence of anti-vaxxers (21, 22). As of today (June 2021), the adult vaccination coverage in Indonesia was still far below the national target (70 vs. 33%; 4.5% of the total population) while vaccination for children were still in talk worldwide (23, 24). Our study shows that parental attitude toward COVID-19 vaccination did not affect their decision on sending their children to school.

Results from the present study showed that both individuals from vulnerable populations and children with comorbidities (asthma, diabetes, and malignancy) are more susceptible to COVID-19 infection (25). Similarly this is a concern for our respondents. As expected, parents with history of positive cases of COVID-19 or death caused by COVID-19 in their community were more likely to keep their children at home. These respondents might be more affected from the impact of COVID-19 compared to those without the experiences. Kroshus reported that disagreement of school reopening was associated with fear of COVID-19 (*B* = 0.19; *p* < 0.001) (20). These findings are in accordance with our results: In our study, the proportion of parents who supported conventional school reopening was higher among those who viewed COVID-19 as a mild disease compared to those who perceived COVID-19 to be a life-threatening ailment.

In addition, supporting materials such as internet access and electronic devices were not always available in rural areas across Indonesia (26). Similar to the findings in our present study, it has previously been found that adequate teacher's training on virtual learning, virtual teacher–student interactions, instructional videos, and electronic worksheets were required for effective virtual classes (27). Moreover, prolonged virtual learning increases digital screen time, which may cause biopsychosocial issues such as reduction of sleeping hours and quality, depression, anxiety symptoms, and social isolation (28). Opportunities to learn verbal and non-verbal social messages and peer learning were also limited. Furthermore, the absence of extracurricular and physical activities was significantly associated with gross motor delay and obesity (28).

Limbers found that mandatory use of masks for children was significantly associated with parents' selecting conventional class (with mandatory masks) instead of a fully virtual class (OR = 2.05; *p* < 0.01; 95% CI: 1.36–3.11) (29). The IPS published a recommendation that children aged 2 years and above wear face masks. Permissible exceptions included children with mental and cognitive disorders and those with chronic heart or lung disease. However, given their developmental stage, younger children cannot be expected to wear masks for a long period of time (14). As shown in Figure 2, opposition to school reopening was more prevalent among parents of children with lower mask-wearing tolerance. Social distancing and limiting the number of students within a classroom could be implemented to overcome this issue.

Our study shows that most parents who had private transportation chose to keep their children at home. In Indonesia, private transportation ownership reflects middle-upper income society, while majority of middle-lower income society use public transportation (30). Although indirectly, this may possibly reflect that those who are socio-economically more advantaged were able to provide a more conducive virtual learning, hence chose to keep their children at home and practice social distancing.

## Conclusions

School reopening is currently an inevitable worldwide challenge for formal education. Challenges faced during online education, including limited interactions between teachers and students, students' mental health problem, absence of caregiver during online learning, and limited supporting education materials must be addressed promptly if online school continues. Many countries with a declining number of confirmed COVID-19 cases are considering reinstatement of conventional learning. However, there are many challenges that must be addressed to prevent new COVID-19 clusters. Combined efforts by all stakeholders, including policymakers, schools, parents, and children, must be implemented and enforced. Although overall knowledge about COVID-19 was found to be sufficient, parents' and children's readiness to return to school is another pivotal determinant that should not be overlooked. In our survey, we found an inconclusive response regarding school reopening among parents with school-aged children in Indonesia. Although parents' knowledge about COVID-19 was adequate, many parents were not confident about sending their child for conventional learning. Thus, we believe that the most important factors for increasing parents' confidence is ensuring that there will be a safe environment in schools. We recommend that school reopening commence only when the health and safety protocols are ready. Increasing the reach of vaccination to the recommended goal *via* proper distribution of vaccines and promoting the efficacy and benefits of vaccination will also increase the preparedness for school reopening.

### Strengths and Limitations

To our knowledge, this is the first national survey about school reopening in Indonesia during the COVID-19 pandemic. The use of social media advertisements allowed our survey to receive nationwide responses. The results of this study could perhaps be extrapolated to other developing countries with a consistently high number of confirmed COVID-19 cases. Our study has several limitations. Firstly, cross-sectional study could not provide a direct causal relationship. In addition, most of our study's respondents were residents of urban areas; thus, the responses to our survey could not accurately represent the rural population. Our respondents were limited to technologically advantaged parents. In addition, we did not record parents' demographic data including education levels, age, and socio-economic status; however, these should be assessed objectively in future studies because they might be critical behavioral determinants for decisions about school reopening. Furthermore, although our questionnaire was approved by The Indonesian COVID-19 Task Force Expert Panel of the IPS approved, we did not perform validity and reliability analysis of the instrument.

## Data Availability Statement

The raw data supporting the conclusions of this article will be made available by the authors, without undue reservation.

## Ethics Statement

The studies involving human participants were reviewed and approved by the Ethics Committee of the Faculty of Medicine, University of Indonesia. The patients/participants provided their written informed consent to participate in this study.

## Author Contributions

AHP, NP, HS, PY, HG, RR, ADP, LN, CS, LH, IU, YP, NK, AA, KK, GH, AT, SA, NS, SW, FA, and ABP designed and provided analysis and interpretation of the data and drafted the manuscript. AHP, NP, HS, NK, and ABP contributed to the conception and design of the manuscript. AHP, NP, HS, PY, HG, RR, ADP, LN, CS, LH, IU, YP, NK, AA, KK, GH, AT, SA, NS, SW, FA, and ABP critically revised the manuscript. All authors provided final approval prior to the submission of the manuscript.

## Conflict of Interest

The authors declare that the research was conducted in the absence of any commercial or financial relationships that could be construed as a potential conflict of interest.

## Publisher's Note

All claims expressed in this article are solely those of the authors and do not necessarily represent those of their affiliated organizations, or those of the publisher, the editors and the reviewers. Any product that may be evaluated in this article, or claim that may be made by its manufacturer, is not guaranteed or endorsed by the publisher.

## References

[1] Cucinotta D Vanelli M. WHO declares COVID-19 a pandemic. Acta Biomed. (2020) 91:157–60. 10.23750/abm.v91i1.9397 32191675 PMC7569573

[2] Yang Y Xiao Z Ye K He X Sun B Qin Z. SARS-CoV-2: characteristics and current advances in research. Virol J. (2020) 17:117. 10.1186/s12985-020-01369-z 32727485 PMC7387805

[3] World Health Organization. Weekly Epidemiological Update on COVID-19 - 8 June 2021. (2021). Available online at: https://www.who.int/publications/m/item/weekly-epidemiological-update-on-covid-19-−8-june-2021 (accessed June 23, 2021).

[4] *Komite Penanganan COVID-19 dan Pemulihan Ekonomi Nasional*. Peta Sebaran COVID-19 (2021). Available online at: https://covid19.go.id/peta-sebaran-covid19 (accessed June 23, 2021).

[5] Djalante R Lassa J Setiamarga D Sudjatma A Indrawan M Haryanto B. Review and analysis of current responses to COVID-19 in Indonesia: period of January to March 2020. Prog Disaster Sci. (2020) 6:100091. 10.1016/j.pdisas.2020.100091 34171011 PMC7149002

[6] Kementerian Kesehatan Republik Indonesia. Surat Edaran Nomor 4 Tahun 2020 Tentang Pelaksanaan Kebijakan Pendidikan Dalam Masa Darurat Penyebaran Virus Corona. (2020). Available online at: https://pusdiklat.kemdikbud.go.id/surat-edaran-mendikbud-no-4-tahun-2020-tentang-pelaksanaan-kebijakan-pendidikan-dalam-masa-darurat-penyebaran-corona-virus-disease-covid-1-9/ (accessed June 23, 2021).

[7] Nugroho RS. Infografik: Pandemi Covid-19, Arti Zona Merah, Oranye, Kuning dan Hijau. (2020). Available online at: https://www.kompas.com/tren/read/2020/06/05/190000065/infografik–pandemi-covid-19-arti-zona-merah-oranye-kuning-dan-hijau (accessed June 23, 2021).

[8] *Komite Penanganan COVID-19 dan Pemulihan Ekonomi Nasional*. Peta Risiko (2021). Available online at: https://covid19.go.id/peta-risiko (accessed June 23, 2021).

[9] Feder G Lucas d'Oliveira AF Rishal P Johnson M. Domestic violence during the pandemic. BMJ. (2021) 372:n722. 10.1136/bmj.n722 33731382

[10] Øverlien C. The COVID-19 pandemic and its impact on children in domestic violence refuges. Child Abuse Rev. (2020) 29:379–86. 10.1002/car.2650 32904999 PMC7461223

[11] Ministry of Education Culture. Sekolah Tatap Muka Terbatas Mulai Juli 2021. (2021). Available online at: https://pmpk.kemdikbud.go.id/read-news/sekolah-tatap-muka-terbatas-mulai-juli-2021 (accessed June 23, 2021).

[12] Indonesian Pediatric Society. Rekomendasi Ikatan Dokter Anak Indonesia Mengenai Pembukaan Sekolah di Masa Pandemi. (2021). Available online at: https://www.idai.or.id/tentang-idai/pernyataan-idai/rekomendasi-ikatan-dokter-anak-indonesia-mengenai-pembukaan-sekolah-di-masa-pandemi (accessed June 23, 2021).

[13] Komite Penanganan COVID-19 dan Pemulihan Ekonomi Nasional. Penyesuaian Kebijakan Pembelajaran di Masa Pendemi COVID-19. (2020). Available online at: https://covid19.go.id/p/protokol/penyesuaian-kebijakan-pembelajaran-di-masa-pandemi-covid-19 (accessed June 23, 2021).

[14] Malik F Marwaha R. Cognitive Development. Treasure Island, FL: StatPearls Publishing (2020). 30725780

[15] Pelikan ER Lüftenegger M Holzer J Korlat S Spiel C Schober B. Learning during COVID-19: the role of self-regulated learning, motivation, and procrastination for perceived competence. Z Erziehwiss. (2021) 24:393–418. 10.1007/s11618-021-01002-x 33686344 PMC7931168

[16] Dong C Cao S Li H. Young children's online learning during COVID-19 pandemic: Chinese parents' beliefs and attitudes. Child Youth Serv Rev. (2020) 118:105440. 10.1016/j.childyouth.2020.105440 32921857 PMC7476883

[17] Cui S Zhang C Wang S Zhang X Wang L Zhamg L. Experiences and attitudes of elementary school students and their parents toward online learning in China during the COVID-19 pandemic: questionnaire study. J Med Internet Res. (2021) 23:e24496. 10.2196/24496 33878022 PMC8136302

[18] Viner RM Bonell C Drake L Jourdan D Davies N Baltag V. Reopening schools during the COVID-19 pandemic: governments must balance the uncertainty and risks of reopening schools against the clear harms associated with prolonged closure. Arch Dis Child. (2021) 106:111–3. 10.1136/archdischild-2020-319963 32747375 PMC7401577

[19] Gandasari D Dwidienawati D. Content analysis of social and economic issues in Indonesia during the COVID-19 pandemic. Heliyon. (2020) 6:e05599. 10.1016/j.heliyon.2020.e05599 33305034 PMC7708812

[20] Kroshus E Hawrilenko M Tandon PS Christakis DA. Plans of US parents regarding school attendance for their children in the fall of 2020: a national survey. JAMA Pediatr. (2020) 174:1–10. 10.1001/jamapediatrics.2020.3864 32797152 PMC7428818

[21] Soares P Rocha JV Moniz M. Factors associated with COVID-19 vaccine hesitancy. Vaccines (Basel). (2021) 9:300. 10.3390/vaccines9030300 33810131 PMC8004673

[22] Ministry of Health Republic of Indonesia. SK Dirjen Nomor HK.02.02/4/1/2021 Tentang Petunjuk Teknis Pelaksanaan Vaksinasi dalam Rangka Penanggulangan Pandemi Covid-19. (2021). Available online at: https://promkes.kemkes.go.id/sk-dirjen-nomor-hk0202412021-tentang-petunjuk-teknis-pelaksanaan-vaksinasi-dalam-rangka-penanggulangan-pandemi-covid19 (accessed June 23, 2021).

[23] Kominfo P. Target Vaksinasi 70% Penduduk, Menkominfo: Butuh Kolaborasi Lebih Masif. (2021). Available online at: https://www.kominfo.go.id/content/detail/35518/siaran-pers-no-238hmkominfo072021-tentang-target-vaksinasi-70-penduduk-menkominfo-butuh-kolaborasi-lebih-masif/0/siaran_pers (accessed June 23, 2021).

[24] Satuan Tugas Penanganan COVID-19. Pengendalian COVID-19 Dengan 3M, 3T, Vaksinasi, Disiplin, Kompak, Dan Konsisten, 2nd Edn. Jakarta: COVID-19 Accelerated Handling Task Force (2021).

[25] Sanyaolu A Okorie C Marinkovic A Patidar R Younis K Desai P. Comorbidity and its impact on patients with COVID-19. SN Compr Clin Med. (2020) 2:1069–76. 10.1007/s42399-020-00363-4 32838147 PMC7314621

[26] The Jakarta Post. State Distance Learning Plan Fails to Account for Poor, Disconnected Students. (2020). Available online at: https://www.thejakartapost.com/news/2020/09/01/state-distance-learning-plan-fails-to-account-for-poor-disconnected-students.html (accessed June 23, 2021).

[27] Almarzooq ZI Lopes M Kochar A. Virtual learning during the COVID-19 pandemic: a disruptive technology in graduate medical education. J Am Coll Cardiol. (2020) 75:2635–8. 10.1016/j.jacc.2020.04.015 32304797 PMC7159871

[28] Stiglic N Viner RM. Effects of screentime on the health and well-being of children and adolescents: a systematic review of reviews. BMJ Open. (2019) 9:e023191. 10.1136/bmjopen-2018-023191 30606703 PMC6326346

[29] Limbers CA. Factors associated with caregiver preferences for children's return to school during the COVID-19 pandemic. J Sch Health. (2021) 91:3–8. 10.1111/josh.12971 33140434

[30] Kusuma CA Multifiah M Syafitri W. Analisis korelasi mobilitas penduduk dan sosioekonomi terhadap kepemilikan kendaraan. Warta Penelitian Perhubungan. (2018) 30:101. 10.25104/warlit.v30i2.830

