# Supplementary material for: Parents' Perspectives Toward School Reopening During COVID-19 Pandemic in Indonesia—A National Survey
Source: Front Public Health. 2022 Apr 4;10:757328. doi: 10.3389/fpubh.2022.757328 (PMC9014259; doi:10.3389/fpubh.2022.757328)
Supplement: Supplementary file 1 [file Data_Sheet_1.PDF]

Supplementary Material 1

Indonesia's zoning guidelines

General regulation

In order to control the movement of the citizen, Indonesia's COVID-19 Accelerated Handling Task Force classified the region into 4 zones (Figure 1), which include green (not affected by COVID-19), yellow (low risk of transmission of COVID-19), orange zone (medium risk of transmission), and red zone (high risk of transmission). Some rules, including the permission to open schools, shops, and offices are controlled according to this zoning.[8]

**Green Zone**<br>1. No positive COVID-19 cases found<br>2. The spread of the Covid-19 virus is under control<br>3. The risk of spread remained only in places for isolation<br>4. Strict and regular monitoring is carried out to prevent potential new cases from arising.	**Yellow Zone**<br>1. The possibility of local transmission is still quite large and may be fast<br>2. Transmission from imported cases may occur quickly<br>3. Household-level transmission may occur<br>4. Controlled cluster of transmission and does not increase
**Orange Zone**<br>1. Local transmission may quickly occur<br>2. Transmission from imported cases may occur quickly<br>3. New clusters must be monitored and controlled through aggressive testing and tracing	**Red Zone**<br>1. There is quick local transmission or spread of the corona virus between local residents in one area<br>2. The epidemic spread widely and many new clusters in the region

Supplementary Figure 1. Characteristics for Area Zoning in Indonesia[8]

Criteria to determine the area zoning include 10 epidemiological indicators, 2 public health surveillance indicators, and 2 health service indicators can be seen in Table 1. The regional risk weighting and zoning are updated weekly. Data of the indicators were taken from the surveillance data and online hospital database by Ministry of Health[9]

Supplementary Table 1. Indicators for area zoning.[9]

**Epidemiological Indicators**
1. Decrease in the number of positive and probable cases during the last week by ≥50% from peak<br>2. Decrease in the number of suspected cases in the last week by ≥50% from peak<br>3. Decrease in the number of deaths of positive and probable cases in the last week by ≥50% from peak<br>4. Decrease in the number of deaths of suspected cases in the last week by ≥50% from peak<br>5. Decrease in the number of positive and probable cases hospitalized in the last week by ≥50% from peak<br>6. Decrease in the number of suspected cases hospitalized in the last week by ≥50% from peak<br>7. Cumulative percentage of recovered cases from all positive and probable cases<br>8. Incidence rate of positive cases per 100,000 population<br>9. Mortality rate of positive cases per 100,000 population<br>10. Incidence rate per 100,000 population

**Public Health Surveillance Indicators**
1. The number of diagnostic sample examinations has increased over the past 2 weeks<br>2. Low positivity rate (target ≤5% positive sample of all tested people)
**Health Service Indicators**
1. The number of beds in the isolation room at the referral hospital is able to accommodate up to >20% of the number of positive COVID-19 patients being treated at the hospital<br>2. The number of beds at the Referral Hospital is able to accommodate up to >20% of the number of suspected, probable, and confirmed COVID-19 patients treated at the hospital
