# Supplementary material for: Parents' Perspectives Toward School Reopening During COVID-19 Pandemic in Indonesia—A National Survey
Source: Front Public Health. 2022 Apr 4;10:757328. doi: 10.3389/fpubh.2022.757328 (PMC9014259; doi:10.3389/fpubh.2022.757328)
Supplement: Supplementary file 2 [file Table_1.pdf]

Supplementary Table 1. Multinomial analysis of factors associated with parents' attitudes toward in-school or virtual learning (n = 17,561).

Variables	Send to school (ref); n (%)	Keep at home; n (%)	Equally likely; n (%)	Send to school vs keep at home		Equally likely vs keep at home		Equally likely vs send to school	
				p value	Adjusted OR (95% CI)	p value	Adjusted OR (95% CI)	p value	Adjusted OR (95% CI)
Vulnerable population in the household				<0·001*	1·18 (1·10 – 1·27)	<0·001*	1·12 (1·00 - 1·26)	0·378	0·95 (0·84 - 1·07)
None (ref)	3,500 (19·9)	2,033 (11·6)	489 (2·8)						
Yes	6,274 (35·7)	4,335 (24·7)	930 (5·3)						
Children with comorbidities				<0·001*	2·56 (2·29 – 2·87)	<0·001*	1·60 (1·33 - 1·94)	<0·001*	0·63 (0·51 - 0·76)
None (ref)	9,186 (52·3)	5,440 (31·0)	1,284 (7·3)						
Yes	588 (3·3)	928 (5·3)	135 (0·8)						
How dangerous is COVID-19?[a]				<0·001*	28·87 (14·29–58·33)	<0·001*	4·00 (1·57 - 10·19)	<0·001*	0·14 (0·07 - 0·26)
Mild (ref)	510 (2·9)	8 (0·0)	10 (0·1)						
Dangerous	9,264 (52·8)	6,360 (36·2)	1,409 (8·0)						
Any positive COVID-19 cases in community				<0·001*	1·75 (1·61 – 1·90)	<0·001*	1·23 (1·07 - 1·43)	<0·001*	0·70 (0·61 - 0·80)
None (ref)	4,253 (24·2)	1,441 (8·2)	442 (2·5)						
Yes	5,521 (29·9)	4,927 (28·1)	977 (5·6)						
Any death caused by COVID-19 in community				<0·001*	2·05 (1·90 – 2·21)	<0·001*	1·43 (1·25 - 1·63)	<0·001*	0·70 (0·61 - 0·80)
None (ref)	6,981 (39·8)	3,004 (17·1)	834 (4·7)						
Yes	2,793 (15·9)	3,364 (19·2)	585 (3·3)						
Agree COVID-19 vaccination for parents				<0·001*	1·69 (1·53 – 1·87)	<0·001*	0·61 (0·48 - 0·77)	0·564	0·56 (0·85 - 1·34)
No (ref)	1,864 (10·6)	635 (3·6)	239 (1·4)						
Yes	7,910 (45·0)	5,733 (32·6)	1180 (6·7)						
Mode of transportation[b]				<0·001*	1·46 (1·30 – 1·66)	<0·001*	0·98 (0·81 – 1·19)	<0·001*	0·66 (0·55 - 0·81)
Public transport (ref)	648 (3·7)	606 (3·5)	137 (0·8)						
Private transport	9,126 (52·0)	5,762 (32·8)	1282 (7·3)						

Notes: ref: reference group. *= significant p-value, [a] "Mild" is score 1-3, while "dangerous" is 4-5 with 5 as the most dangerous score. [b] Public transport include online transport and mass public transport, while private transport nclude private car, private motorbike, walking, and cycling.

Supplementary Table 2. Multivariate analysis of factors associated with parents' attitudes combining equally likely with decision of in-school vs online learning (n = 17,561).

Variables	Send to school (ref); n (%)	Keep at home; n (%)	Equally likely; n (%)	Send to school + equally likely vs keep at home		Send to school vs equally likely + keep at home	
				p value	Adjusted OR (95% CI)	p value	Adjusted OR (95% CI)
Vulnerable population in the household				<0·001*	1·17 (1·09 - 1·25)	<0·001*	1·15 (1·08-1·23)
None (ref)	3,500 (19·9)	2,033 (11·6)	489 (2·8)				
Yes	6,274 (35·7)	4,335 (24·7)	930 (5·3)				
Children with comorbidities				<0·001*	2·37 (2·13 - 2·64)	<0·001*	2·37 (2·12 - 2·65)
None (ref)	9,186 (52·3)	5,440 (31·0)	1,284 (7·3)				
Yes	588 (3·3)	928 (5·3)	135 (0·8)				
How dangerous is COVID-19?[a]				<0·001*	25·39 (12·58 - 51·25)	<0·001*	16·81 (10·46 - 27·04)
Mild (ref)							
Dangerous	510 (2·9)	8 (0·0)	10 (0·1)				
	9,264 (52·8)	6,360 (36·2)	1,409 (8·0)				
Any positive COVID-19 cases in community				<0·001*	1·68 (1·55 - 1·82)	<0·001*	1·68 (1·56 - 1·81)
None (ref)	4,253 (24·2)	1,441 (8·2)	442 (2·5)				
Yes	5,521 (29·9)	4,927 (28·1)	977 (5·6)				
Any death caused by COVID-19 in community				<0·001*	1·93 (1·80 – 2·08)	<0·001*	1·92 (1·78 - 2·06)
None (ref)	6,981 (39·8)	3,004 (17·1)	834 (4·7)				
Yes	2,793 (15·9)	3,364 (19·2)	585 (3·3)				
Agree COVID-19 vaccination for parents				<0·001*	1·69 (1·53 - 1·87)	<0·001*	1·48 (1·35 - 1·62)
No (ref)	1,864 (10·6)	635 (3·6)	239 (1·4)				
Yes	7,910 (45·0)	5,733 (32·6)	1180 (6·7)				
Mode of transportation[b]				<0·001*	1·38 (1·23 - 1·55)	<0·001*	1·49 (1·32 – 1·67)
Public transport (ref)	648 (3·7)	606 (3·5)	137 (0·8)				
Private transport	9,126 (52·0)	5,762 (32·8)	1282 (7·3)				

Notes: ref: reference group. *= significant p-value, [a] "Mild" is score 1-3, while "dangerous" is 4-5 with 5 as the most dangerous score. [b] Public transport include online transport and mass public transport, while private transport nclude private car, private motorbike, walking, and cycling.
